# Penetrating Wound of the Heart

**Published:** 1861-09

**Authors:** 


### PENETRATING WOUND OF THE HEART.

CASE IN WHICH DEATH TOOK PLACE TEN YEARS AFTER. By *Dr. Mühlig.*—Dr. Mühlig states that as far as he is aware only three cases (related by Velpeau, Laugier, and Latour of Orleans) are on record in which life has been prolonged for some years after a wound of the heart. He therefore relates the following remarkable case. The subject of it, a mason, ten years before he came under the author's notice, had received a blow with a stiletto on the left side of the sternum, the cicatrix left after the healing of the wound being there still visible. Although the man's life continued awhile in danger, he recovered so well as to be enabled to follow his employment without any inconvenience, always, however, perceiving a bellows-sound in the region of his heart. He came to the hospital with alarming symptoms only a few weeks prior to his death, excessive dyspnœa, anasarca, and a very intense bellows-sound being present. This last was a double bruit, accompanying the systole and diastole, and entirely obscuring the physiological signs. The man got worse and worse, his end being hastened by the supervention of gangrene. At the autopsy the left lung was found connected throughout its entire surface to the thorax and pericardium by means of old adhesions. On cutting into the pericardium it was found to be intimately adherent to the heart throughout its whole extent, so as to require strong traction for its removal. On the side of the right ventricle it could not be removed without cutting, and here was felt a hard, prominent body, covered by the sternum, and which at first was believed to be indurated fibrin. The heart was considerably increased in size, its hypertrophy principally implicating the left ventricle, the cavity of which was also enlarged and elongated. The right ventricle seemed to form only an appendix, and on the inner surface of its wall a rounded aperture, lined with cicatricial tissue, was observed, through which the little finger could be passed into a sac about the size of a small walnut. At the bottom of this was felt a hard body covered with asperities, giving very much the sensation imparted by a carious bone. This was the same indurated body which had been felt through the pericardium. On opening the sac it was found to be a partial aneurism of the heart, containing an incrusted fibrinous concretion, and having its wall solely constituted of the two layers of the pericardium fused into one, no trace of muscular fibres being present. The aneurism was situated at the union of the two upper thirds with the lower third of the wall of the right ventricle; and exactly opposite the aneurism there was a hole in the intraventricular septum leading into the left ventricle, which scarcely admitted the point of the little finger. But on the side of the left ventricle this hole was twice as wide, and it was completely lined with cicatricial tissue, which extended several lines into the left ventricle, forming above a semilunar fold which partially closed the orifice, a similar fold below floating freely in the cavity of the ventricle. Close to the free edge of each semilunar valve of the aorta was a cauliflower excrescence, larger than the size of a pea, and viewed from the cavity of the aorta the valves exhibited a solution of continuity near their free edges, a probe passing through this into the middle of the fungosities. The substance of the heart was of a pale red, with yellowish streaks, and it was flaccid and friable. The auricles were much dilated, and their walls were thinned.—(*Presse Médicale Belge*, 1860, No. 43; *Med. Times and Gazette.*)

